# Cytogenetic Analysis of the Members of the Snake Genera *Cylindrophis*, *Eryx*, *Python*, and *Tropidophis*

**DOI:** 10.3390/genes13071185

**Published:** 2022-07-01

**Authors:** Tomáš Charvát, Barbora Augstenová, Daniel Frynta, Lukáš Kratochvíl, Michail Rovatsos

**Affiliations:** 1 Department of Ecology, Faculty of Science, Charles University, Viničná 7, 12844 Prague, Czech Republic; tomas.charvat@natur.cuni.cz (T.C.); augstenova.barbora@gmail.com (B.A.); kratoch1@natur.cuni.cz (L.K.); 2 Department of Zoology, Faculty of Science, Charles University, Viničná 7, 12844 Prague, Czech Republic; frynta@centrum.cz

**Keywords:** boa, C-banding, CGH, evolution, FISH, heterochromatin, karyotype, python, rDNA, sex chromosomes, telomeres

## Abstract

The recent discovery of two independently evolved XX/XY sex determination systems in the snake genera *Python* and *Boa* sparked a new drive to study the evolution of sex chromosomes in poorly studied lineages of snakes, where female heterogamety was previously assumed. Therefore, we examined seven species from the genera *Eryx*, *Cylindrophis*, *Python*, and *Tropidophis* by conventional and molecular cytogenetic methods. Despite the fact that these species have similar karyotypes in terms of chromosome number and morphology, we detected variability in the distribution of heterochromatin, telomeric repeats, and rDNA loci. Heterochromatic blocks were mainly detected in the centromeric regions in all species, although accumulations were detected in pericentromeric and telomeric regions in a few macrochromosomes in several of the studied species. All species show the expected topology of telomeric repeats at the edge of all chromosomes, with the exception of *Eryx muelleri*, where additional accumulations were detected in the centromeres of three pairs of macrochromosomes. The rDNA loci accumulate in one pair of microchromosomes in all *Eryx* species and in *Cylindrophis ruffus*, in one macrochromosome pair in *Tropidophis melanurus* and in two pairs of microchromosomes in *Python regius*. Sex-specific differences were not detected, suggesting that these species likely have homomorphic, poorly differentiated sex chromosomes.

## 1. Introduction

Snakes (Serpentes) are a diverse group of squamate reptiles, with approximately 3970 species, representing roughly one-third of the total reptile species diversity [1]. The majority of extant species of snake belong to the group Caenophidia (3283 species), while the rest are divided into two major groups: Henophidia (228 species, mainly boas and pythons) and Scolecophidia (466 species, commonly known as blind snakes or thread snakes) [1]. These two groups are, however, mostly recognized due to historical reasons, as they are paraphyletic according to recent phylogenetic reconstructions [2,3,4,5]. Despite their striking diversity, snakes have quite conserved karyotypes. Although their diploid chromosome number varies between 2n = 24 and 2n = 56 [6,7,8], the most common chromosome number found in the majority of snake lineages is 2n = 36, with 16 macrochromosomes and 20 microchromosomes [7,8].

So far, only genotypic sex determination has been documented in snakes [9]. While homologous, highly differentiated and often heteromorphic ZZ/ZW sex chromosomes or derived systems of multiple sex chromosomes were reported in all examined caenophidian species [10,11,12,13,14,15], we still have limited knowledge on the sex chromosome evolution in henophidian and scolecophidian snakes. Heteromorphic ZZ/ZW sex chromosomes were detected in the henophidian Madagascar boa *Acrantophis* sp. cf. *dumerili* (Sanziniidae) [13,16] and, very recently, in the scolecophidian *Myriopholis macrorhyncha* [17]. Nevertheless, cytogenetic analyses did not reveal sex chromosomes in other henophidian or scolecophidian species, and in many older studies, only one sex was examined [8,13,18,19,20,21,22,23]. Furthermore, sex chromosomes were not detected in *Boa constrictor*, neither by comparative read depth (genome coverage) analysis nor by Illumina reads between sexes [24]. For decades, it was speculated that all snakes might have homologous ZZ/ZW sex chromosomes, which are heteromorphic and highly differentiated in caenophidian snakes but homomorphic and poorly differentiated in henophidian and scolecophidian snakes [20,21,24,25,26]. However, this view was proved incorrect when two non-homologous XX/XY systems were detected in a python (*Python bivittatus*; Pythonidae) and two species of boa (*Boa constrictor*, *B. imperator*; Boidae) by single-nucleotide polymorphism (SNP) analysis of RAD-seq genomic data [27]. Notably, the sex chromosomes of *P. bivittatus* are partially homologous to ZZ/ZW sex chromosomes of caenophidian snakes, while the sex chromosomes in the two boas are homologous to an autosome of caenophidian snakes. These recent cytogenetic and genomic findings have revived the interest of the scientific community to further explore the evolution of sex chromosomes in snakes.

In the present study, we cytogenetically examined seven henophidian species: the sand boas *Eryx colubrinus*, *E. conicus*, *E. miliaris,* and *E. muelleri* (Erycidae); the ball python *Python regius* (Pythonidae); the red-tailed pipe snake *Cylindrophis ruffus* (Cylindrophiidae); and the Cuban wood snake *Tropidophis melanurus* (Tropidophiidae). Our aim was to expand our knowledge on the karyotypic traits of these species, with emphasis on exploring the presence of sex chromosomes. The selected species have a potentially phylogenetically informative position for the reconstruction of sex chromosome evolution in snakes. *T. melanurus* is a member of the lineage Amerophidia and sister to all other henophidian and caenophidian snakes [2,5]. The ball python *P. regius* is closely related to *P. bivittatus*, a species with an XX/XY sex determination system [27], and *C. ruffus* is a member of the lineage relatively closely related to pythons [2,5]. The sand boas of the genus *Eryx* are phylogenetically nested between lineages of boas with documented ZZ/ZW (*A.* sp. cf. *dumerili*) and XX/XY (*B. constrictor*, *B. imperator*) sex chromosomes. We applied conventional (karyotype reconstruction and C-banding) and molecular (in situ hybridization with probes for telomeric repeats and rDNA loci and comparative genome hybridization) cytogenetic methods. The presence of telomeric repeats in the interstitial parts of chromosomes might help uncover cryptic chromosomal rearrangements and further expand the knowledge of karyotype evolution [28]. Furthermore, heterochromatin, rDNA loci, and telomeric repeats tend to accumulate on reptile sex chromosomes [15,29,30,31,32,33], and may be suitable markers for identifying homomorphic sex chromosomes.

## 2. Materials and Methods

### 2.1. Samples and Species Verification

Seven non-caenophidian snake species were used for this study. We examined specimens of both sexes of *Eryx colubrinus*, *E. conicus*, *E. miliaris, E. muelleri* (Erycidae), and *Python regius* (Pythonidae), but only female specimens of *Cylindrophis ruffus* (Cylindrophiidae) and *Tropidophis melanurus* (Tropidophiidae) (Table 1). All animals were obtained from the pet trade and were either captive-bred or legally imported from the wild. Blood samples were collected from each individual, and were used for extraction of DNA and leukocyte cultivation for preparation of chromosome suspensions.

Because taxon identification based on external morphology can often be unreliable, and taxonomic nomenclature is occasionally revised in snakes, we chose to provide the sequences of the mitochondrial loci cytochrome b (*cytb*) and cytochrome c oxidase subunit I (*coi*) as a genetic identity of the individuals that we cytogenetically examined (see Rovatsos et al. [34]). For this task, DNA was isolated from fresh blood samples using a DNeasy Blood and Tissue Kit (Qiagen, Hilden, Germany). To amplify the desirable mitochondrial regions, we used primers RepCOI-F/RepCOI-R [35] and L14919/H16064 [36,37] for *coi* and *cytb*, respectively. The parameters of the PCR and amplification conditions were previously reported in Koubová et al. [38] and Mazzoleni et al. [39]. The PCR products were sent for bi-directional Sanger sequencing to Macrogen (Korea). The sequences were trimmed and aligned in Geneious Prime v.2022.1.1 (https://www.geneious.com, accessed on 6 May 2022) and compared with other available sequences in the GenBank database using BLAST [40].

### 2.2. Chromosome Preparation and Staining

Chromosome suspensions were obtained via leukocyte cultivation from fresh whole-blood samples as in Mazzoleni et al. [39]. Slides with chromosome spreads were stained by Giemsa for evaluation of the quality of the chromosome suspension and for karyogram reconstruction. Karyograms were reconstructed using the Ikaros karyotyping system (MetaSystems, Altlussheim, Germany).

The topology of constitutive heterochromatin was visualized using the standard C-banding protocol [41] but Fluoroshield with DAPI (Vector Laboratories, Burlingame, CA, USA), instead of Giemsa, was used to stain the chromosomal material. The studied species differed in the minimal BaOH_2_ treatment time needed to sufficiently visualize the heterochromatic blocks: 5 min for *Eryx colubrinus* and *Cylindrophis ruffus*, 8 min for *Python regius*, 10 min for *E. miliaris*, 15 min for *E. conicus*, and 18 min for *E. muelleri* and *Tropidophis melanurus*.

### 2.3. Fluorescence In Situ Hybridization (FISH) with Probes for Repetitive Elements

FISH with a telomeric probe was performed to visualize the distribution of telomeric-like sequences and, moreover, to uncover putative interstitial telomeric repeats. The probe with the (TTAGGG)_n_ motif was prepared by PCR without a template, according to our published protocol (Rovatsos et al. [34], based on Ijdo et al. [42]). Plasmid (pDmr.a 51#1) with an 11.5 kb insert encoding the 18S and 28S ribosomal units of *Drosophila melanogaster* [43] was used for rDNA probe preparation. It was cut to 200–300 bp long fragments and labeled with dUTP-biotin by nick translation (Abbott Molecular, Des Plaines, IL, USA). Probes were precipitated using salmon sperm, sodium acetate (3M), and 96% ice-cold ethanol, and resuspended in hybridization buffer (50% formamide in 2 × SSC). The treatment of the chromosome suspensions and the probe, the hybridization conditions, the post-hybridization washes, and the signal amplification were performed following the protocols from Rovatsos et al. [28] and Mazzoleni et al. [39].

### 2.4. Comparative Genome Hybridization (CGH)

DNA samples from males were labeled with dUTP-biotin (Roche, Basel, Switzerland), while DNA samples from females were labeled with dUTP-digoxigenin (Roche, Basel, Switzerland) using nick translation (Abbott Laboratories, Lake Bluff, IL, USA), according to the manufacturer’s protocol. Labeled DNA samples from a male and a female specimen of the same species were mixed in equal concentration, purified by ethanol precipitation, and resuspended in hybridization buffer (50% formamide in 2 × SSC). The treatment of chromosome suspensions and probes, the hybridization conditions, the post-hybridization washes and the signal detection were performed following the protocol from Rovatsos et al. [44].

### 2.5. Microscopy Analysis

Giemsa-stained metaphases were captured on a Zeiss Axio Imager Z2 microscope equipped with an automatic Metafer-MSearch scanning platform and a CoolCube 1 b/w digital camera (MetaSystems, Altlussheim, Germany). Metaphases stained with C-banding and in situ hybridization techniques were captured with a Provis AX70 fluorescence microscope equipped with a DP30BW digital camera (Olympus, Tokyo, Japan). All images were acquired in black and white, and later processed using DP Manager imaging software (Olympus, Tokyo, Japan).

## 3. Results

### 3.1. Karyotype Reconstruction

**Figure 1:**
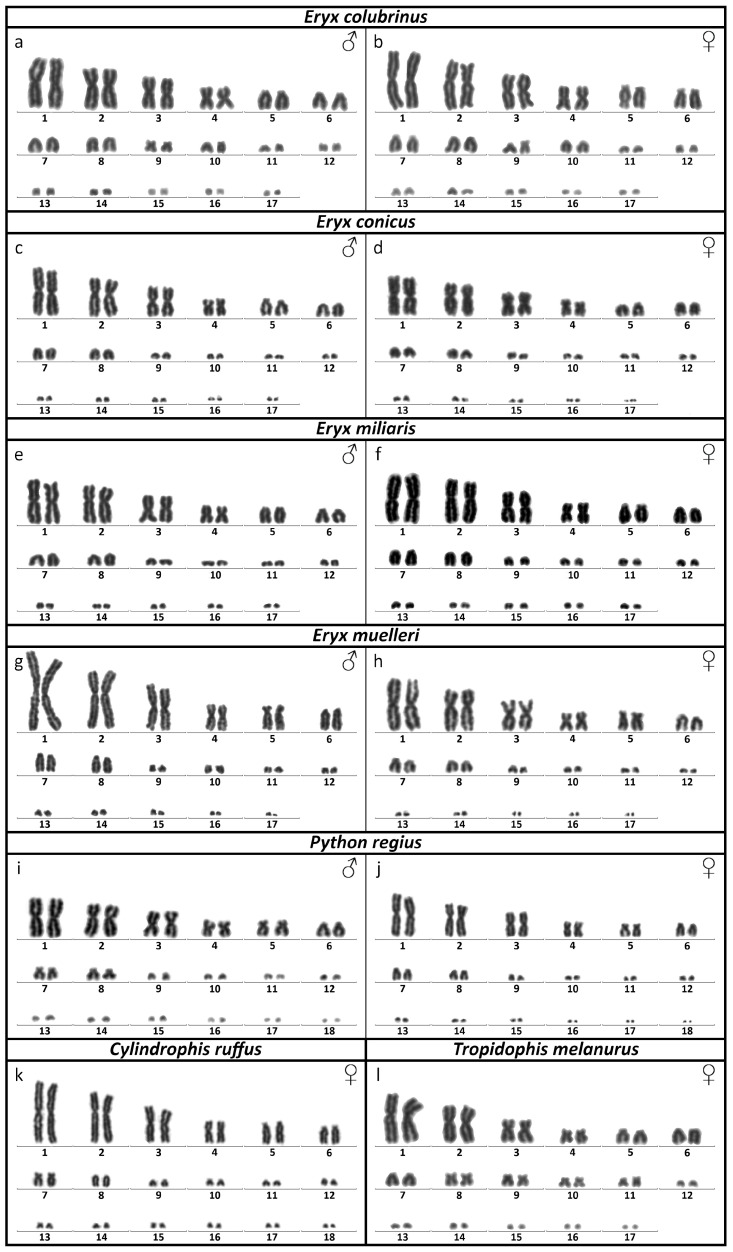
Giemsa-stained karyograms are depicted for the species: (**a**,**b**) *Eryx colubrinus*, (**c**,**d**) *Eryx conicus*, (**e**,**f**) *Eryx miliaris*, (**g**,**h**) *Eryx muelleri*, (**i**,**j**) *Python regius*, (**k**) *Cylindrophis ruffus*, and (**l**) *Tropidophis melanurus*. Sex is indicated.

All four tested species of sand boas have karyotypes with 2n = 34 chromosomes. *Eryx conicus*, *E. muelleri*, and *E. miliaris* have karyotypes with 16 macrochromosomes and 18 microchromosomes and share similar chromosome morphology. Pairs 1, 3, and 4 are metacentric, while pair 2 is submetacentric, and the remaining macrochromosomes are acrocentric. The morphology of the microchromosomes was not distinguishable (Figure 1c–h). *E. colubrinus* has 20 macrochromosomes and 14 microchromosomes. Chromosome pairs 9 and 10, which are microchromosomes in other sand boas, are much larger in this species. Chromosome pair 9 is submetacentric, while pair 10 is acrocentric (Figure 1a,b).

*Tropidophis melanurus* also has a diploid chromosome number of 2n = 34, with 22 macrochromosomes and 12 microchromosomes. Pairs 1–4 are submetacentric, and the remaining macrochromosome pairs are acrocentric (Figure 1l).

The karyotypes of *Cylindrophis ruffus* and *Python regius* have 2n = 36 chromosomes, with 16 macrochromosomes and 20 microchromosomes. Pairs 1, 3, and 4 are metacentric, pair 2 is submetacentric, while pairs 5–8 are acrocentric (Figure 1i–k).

Heteromorphic sex chromosomes were not detected in any of the tested snake species.

### 3.2. C-Banding

**Figure 2:**
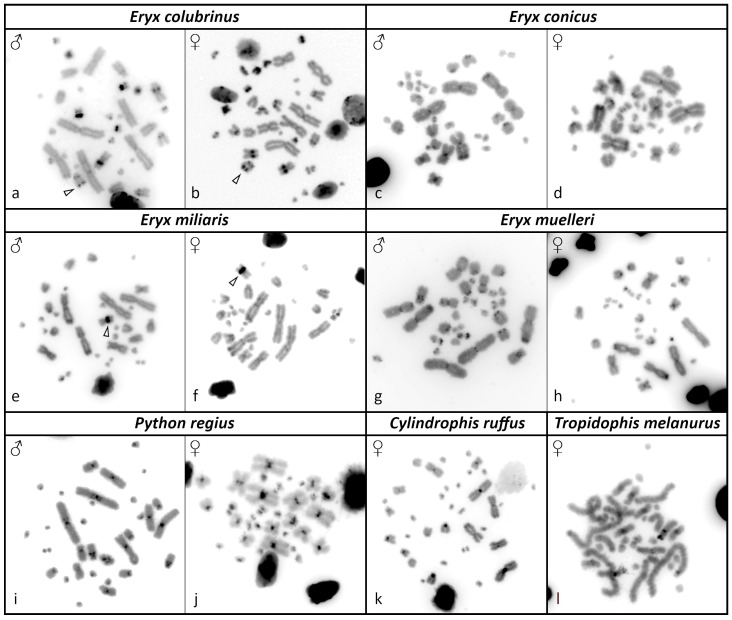
C-banded metaphases for the species: (**a**,**b**) *Eryx colubrinus*, (**c**,**d**) *Eryx conicus*, (**e**,**f**) *Eryx miliaris*, (**g**,**h**) *Eryx muelleri*, (**i**,**j**) *Python regius*, (**k**) *Cylindrophis ruffus*, and (**l**) *Tropidophis melanurus*. Chromosomes with polymorphism in heterochromatic blocks are indicated by arrowheads. Sex is indicated.

In *Python regius*, constitutive heterochromatin is located in the centromeric region of all chromosomes and in the pericentromeric region of chromosome pairs 1 and 6 (Figure 2i,j). In addition to centromeric heterochromatin, all sand boas and *Cylindrophis ruffus* have heterochromatic blocks in the terminal region of the q-arm of the second largest chromosome pair (Figure 2a–h,k). A similar signal in the first largest chromosome pair is present in *Tropidophis melanurus* (Figure 2l). Furthermore, *Tropidophis melanurus* has an extensive accumulation of heterochromatin at the centromere and at the interstitial position of the sixth macrochromosome pair (Figure 2l).

We detected intraspecific heterochromatin heteromorphism in both males and females of two sand boa species. A large heterochromatin block was found in the pericentric region of one chromosome from the fourth largest pair in a male and a female of *Eryx miliaris* (Figure 2e,f), which is missing in the other four conspecific individuals in both sexes. On the other hand, all four studied individuals of *E. colubrinus* display heterochromatin heteromorphism in the telomeric region of the q-arm on one chromosome from the seventh pair. This species has heterochromatin blocks in the pericentromeric region on the q-arms of chromosome pairs 4, 5, 6 and 8. Chromosome pairs 9 and 10 are highly heterochromatic (Figure 2a,b).

Sex-specific differences in the heterochromatin distribution were not detected in any of the examined species.

### 3.3. Fluorescence In Situ Hybridization

**Figure 3:**
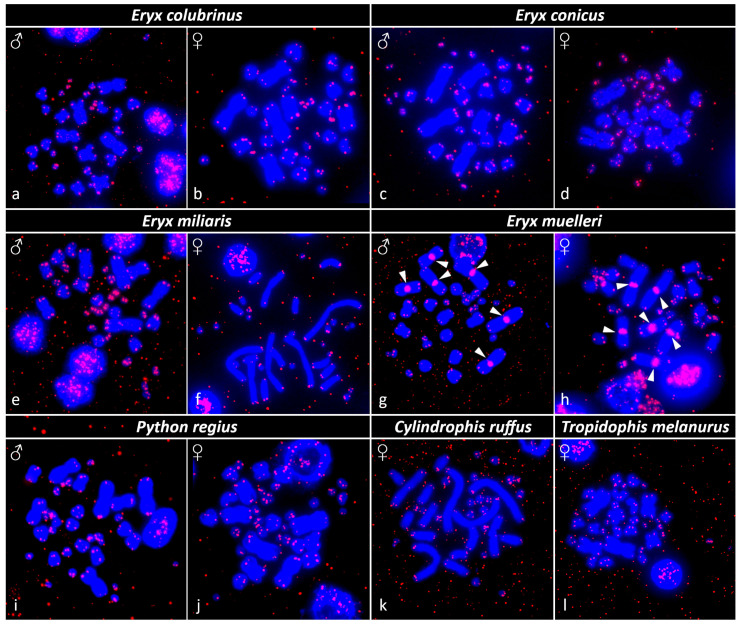
The distribution of telomeric repeats (TTAGGG)_n_ in metaphases. (**a**,**b**) *Eryx colubrinus*, (**c**,**d**) *Eryx conicus*, (**e**,**f**) *Eryx miliaris*, (**g**,**h**) *Eryx muelleri*, (**i**,**j**) *Python regius*, (**k**) *Cylindrophis ruffus,* and (**l**) *Tropidophis melanurus*. Chromosomes with ITRs are marked with arrowheads. Sex is indicated.

The signal from FISH with the telomeric probe was detected in the expected terminal regions of all chromosomes in all tested species (Figure 3). In addition, *Eryx muelleri* has interstitial telomeric repeats (ITRs) in the centromeric region of the first three largest chromosome pairs (Figure 3g,h).

**Figure 4:**
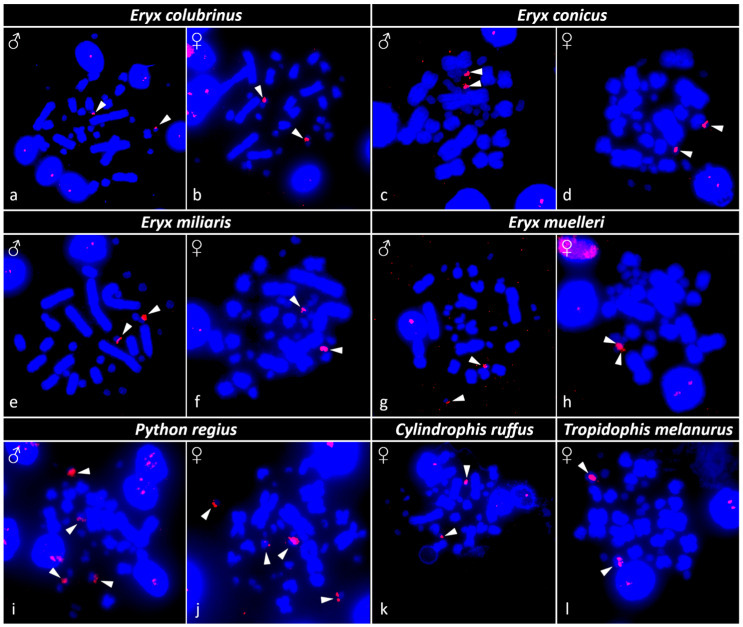
The distribution of rDNA loci in metaphases for the species: (**a**,**b**) *Eryx colubrinus*, (**c**,**d**) *Eryx conicus*, (**e**,**f**) *Eryx miliaris*, (**g**,**h**) *Eryx muelleri*, (**i**,**j**) *Python regius*, (**k**) *Cylindrophis ruffus*, and (**l**) *Tropidophis melanurus*, marked with arrowheads. Sex is indicated.

The rDNA loci are located on one macrochromosome pair in *Tropidophis melanurus*; one microchromosome pair in *Eryx colubrinus*, *E. conicus*, *E. miliaris*, *E. muelleri*, and *Cylindrophis ruffus;* and on two microchromosome pairs in *Python regius* (Figure 4). Sex-specific differences were not detected in the topology of rDNA loci or telomeric repeats in any of the studied species.

### 3.4. Comparative Genome Hybridization

**Figure 5:**
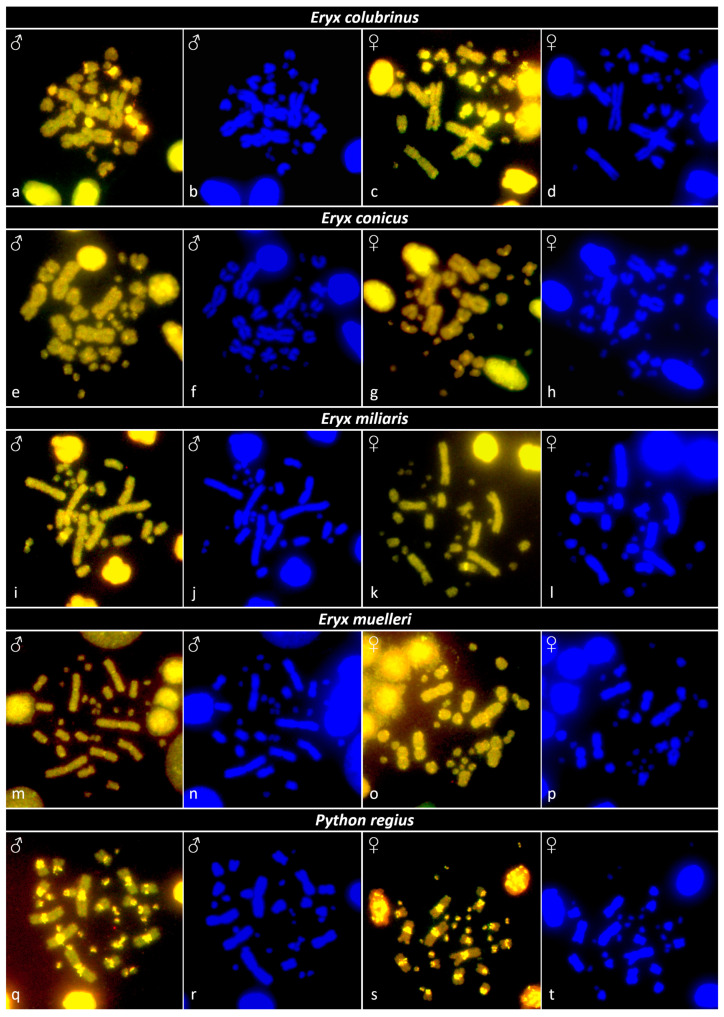
Comparative genome hybridization in metaphases for the species: (**a**–**d**) *Eryx colubrinus*, (**e**–**h**) *Eryx conicus*, (**i**–**l**) *Eryx miliaris*, (**m**–**p**) *Eryx muelleri*, and (**q**–**t**) *Python regius*. Sex-specific regions were not identified in any of the studied specimens. For each metaphase, we present an image of the merged signal from the hybridization of the green (male-specific) and red (female-specific) probe and a photo with DAPI stain to better visualize the chromosome morphology. Sex is indicated.

CGH experiments were performed for all tested species from the genus *Eryx* and for the species *Python regius*, where DNA and chromosome suspensions were available from both sexes. However, sex-specific differences were not detected in these species (Figure 5).

## 4. Discussion

To the best of our knowledge, the karyotypes of five out of seven included snake species were presented here for the first time, specifically for *Eryx colubrinus*, *E. miliaris, E. muelleri*, *Python regius*, and *Tropidophis melanurus*.

In accordance with previous studies [11,45,46], we conclude that all species of sand boas so far examined share a diploid chromosome number of 2n = 34, which is possibly an apomorphy of Erycidae, as species from closely related groups have mostly 2n = 36 chromosomes [7,13,23,47,48]. However, while all other sand boas have 16 macrochromosomes and 18 microchromosomes and share chromosome morphology, *E. colubrinus* has 20 macrochromosomes and 14 microchromosomes. It is likely that two pairs of former microchromosomes increased in size in this species, as the morphology of other chromosomes is otherwise shared with the rest of the sand boas. Both of these pairs also contain large heterochromatic blocks, which likely play a role in the aforementioned size difference, either by amplification of repetitive elements or translocation from another chromosome and further heterochromatinization.

Polymorphism in the distribution of heterochromatin was found in all tested individuals of *E. colubrinus* and in two out of six studied individuals of *E. miliaris*. Heterochromatin heteromorphism was previously described in numerous species of vertebrates [49,50,51,52,53]. Notably, in several species of newt of the genus *Triturus*, all individuals are heterozygous in certain chromatin blocks, as homozygosity in them is lethal [54]. A similar case of heterochromatin heteromorphism was recently documented in Malagasy tomato frogs from the genus *Dyscophus* [55].

*T. melanurus* has a diploid chromosome number of 2n = 34 (22 macrochromosomes and 12 microchromosomes), which is surprising as the only other member of the family Tropidophiidae with the reported chromosome number has 2n = 26 [56] in [8]. Such variability in diploid chromosome numbers is rare in snakes, and it has been reported in a few lineages, such as the tree boas of the genus *Corallus* [22] and the Malagasy snakes of the subfamily Pseudoxyrhophiinae [57], so further examination of additional species from the family Tropidophiidae may help us to better understand the karyotype evolution in this group.

rDNA loci typically accumulate in a single pair of microchromosomes in henophidian snakes [22,58,59,60], except for *Candoia paulsoni*, which has an additional accumulation on a second pair of macrochromosomes [23]. All examined species of the genus *Eryx* as well as *C. ruffus* show clusters of rDNA loci on one pair of microchromosomes. However, we uncovered the presence of rDNA loci on two microchromosome pairs in *Python regius*, even though such a pattern has not been described in other pythons and despite their generally conserved karyotypes [17,23,58]. On the contrary, rDNA loci seem to accumulate on one pair of macrochromosomes in *T. melanurus*. Such cases of rDNA loci accumulation in macrochromosomes have been reported in a few species of caenophidian and scolecophidian snakes [17,58,61], which can be explained either by (i) chromosome fusion of the ancestral rDNA loci-carrying microchromosome with a macrochromosome, or (ii) translocation of rDNA loci to a macrochromosome.

We detected ITRs in the centromeric region of macrochromosome pairs 1–3 in *E. muelleri* but not in other sand boas. Considering that the chromosome morphology of this species is shared with other sand boas (except for *E. colubrinus,* as mentioned above), we suppose that ITRs in this species are likely an outcome of cryptic intrachromosomal rearrangements, such as inversions. Furthermore, the centromeric satellite content is very dynamic, and even closely related species might have a different composition of repeats [23,62,63,64]. FISH with telomeric probes did not detect ITRs in *T. melanurus;* however, the presence of the interstitial heterochromatin on the sixth chromosome pair might suggest a possible fusion point (e.g., tandem fusion), which might also explain the lower chromosome number (2n = 34) in this species compared with the typical snake karyotype (2n = 36). We conclude that the distribution of ITRs and rDNA loci, although generally stable on a larger scale, might be quite variable even among closely related snake species despite similarities in chromosome morphology [17,23,65]. Notably, the intense signal of telomeric repeats was detected in the majority of the microchromosomes in snakes of the genus *Eryx* (Figure 3). These microchromosomes are tiny and dot-like; therefore, we cannot safely conclude whether the intense signal derives from ITRs or the extended arrays of terminal telomeres. A similar pattern has been identified in other reptilian species, such as the dragonsnake *Xenodermus javanicus* [66], monitors, and helodermatids [28,63]. One potential explanation is that microchromosomes often have higher recombination rates than autosomes in vertebrates, including birds and snakes [67,68,69]. We speculate that the repair of the breaks occurring in DNA strands during recombination might lead to the incorporation of telomeric repeats, as telomerase, the enzyme that synthesizes telomeric sequences, is often involved in DNA repair [70]. The evolutionary or functional significance of a higher number of telomeric copies in microchromosomes is not fully understood and deserves further investigation in the future.

Although the methods used in this study had proved effective for uncovering sex chromosomes in some squamate lineages in the past, they did not reveal any sex-specific differences in the examined snake species. This is true even for *Python* *regius*, where X and Y sex chromosomes are expected due to the observed pattern of inheritance of a partially sex-linked phenotypic trait [71] and for which there are reports of facultative parthenogenesis, which leads to all-female offspring [72]. The Madagascar boa *A.* sp. cf. *dumerili* remains the only henophidian snake with detected heteromorphic sex chromosomes. This snake seems to have evolved heteromorphic sex chromosomes by an inversion, but its Z and W are probably poorly differentiated at the sequence level, as CGH did not reveal any female-specific pattern on its W chromosome [16]. Thus, we can conclude that all tested *Eryx* species and *P. regius* have poorly differentiated sex chromosomes similar to almost all of the other studied henophidian snakes. Male individuals of *C. ruffus* and *T. melanurus* should be examined in the future to investigate the possible presence of heteromorphic sex chromosomes. However, we cannot rule out that environmental sex determination might also be present in some henophidian snakes, although it has not yet been reported in any snake [9]. Poorly differentiated sex chromosomes are more prone to turnovers than highly differentiated sex chromosomes [73], which can—together with differences in lineage ages—explain the emerging pattern of the higher variability in sex chromosome systems in snakes from the scolecophidian and henophidian groups compared with the evolutionary stable ZZ/ZW sex chromosomes of caenophidian snakes [14,16,24,27]. Molecular methods such as RAD-seq or whole-genome coverage analyses have been successful in uncovering sex determination systems not only in snakes but also in other squamate lineages with poorly differentiated sex chromosomes [27,74,75,76], and might offer a way to resolve sex determination systems in scolecophidian and henophidian snakes in the future.

## Figures and Tables

**Table 1:** List of examined specimens.

Family	Species	Sex	
		♂	♀
Cylindrophiidae	*Cylindrophis ruffus*	0	1
Erycidae	*Eryx colubrinus*	2	2
	*Eryx conicus*	1	1
	*Eryx miliaris*	3	3
	*Eryx muelleri*	1	1
Pythonidae	*Python regius*	3	4
Tropidophiidae	*Tropidophis melanurus*	0	1

## Data Availability

Not applicable.
